# Incidence of rectal cancer after colectomy for inflammatory bowel disease: nationwide study

**DOI:** 10.1093/bjsopen/zrae074

**Published:** 2024-10-15

**Authors:** Mohammed Deputy, Guy Worley, Elaine M Burns, Alex Bottle, Paul Aylin, Ailsa Hart, Omar Faiz

**Affiliations:** Surgical Epidemiology, Trials and Outcome Centre, St Mark’s Hospital and Academic Institute, Harrow, UK; Department of Surgery and Cancer, Imperial College London, London, UK; Surgical Epidemiology, Trials and Outcome Centre, St Mark’s Hospital and Academic Institute, Harrow, UK; Department of Surgery and Cancer, Imperial College London, London, UK; Surgical Epidemiology, Trials and Outcome Centre, St Mark’s Hospital and Academic Institute, Harrow, UK; Department of Surgery and Cancer, Imperial College London, London, UK; School of Public Health, Imperial College London, London, UK; School of Public Health, Imperial College London, London, UK; Department of Surgery and Cancer, Imperial College London, London, UK; Department of Gastroenterology, St Mark’s Hospital and Academic Institute, Harrow, UK; Surgical Epidemiology, Trials and Outcome Centre, St Mark’s Hospital and Academic Institute, Harrow, UK; Department of Surgery and Cancer, Imperial College London, London, UK

## Abstract

**Background:**

Inflammatory bowel disease increases the risk of colorectal neoplasia. A particular problem arises in patients who have undergone subtotal colectomy leaving a rectal remnant. The risk of future rectal cancer must be accurately estimated and weighed against the risks of further surgery or surveillance. The aim of this study was to estimate the 10-year cumulative incidence of rectal cancer in such patients.

**Methods:**

A nationwide study using England’s hospital administrative data was performed. A cohort of patients undergoing subtotal colectomy between April 2002 and March 2014 was identified. A competing risks survival analysis was performed to calculate the cumulative incidence of rectal cancer. The effect of the COVID-19 pandemic on endoscopic surveillance was investigated using time-trend analysis.

**Results:**

A total of 8120 patients were included and 61 patients (0.8%) were diagnosed with cancer. The cumulative incidence of rectal cancer was 0.26% (95% c.i. 0.17% to 0.39%), 0.49% (95% c.i. 0.36% to 0.68%), and 0.77% (95% c.i. 0.57% to 1.02%) at 5, 10, and 15 years respectively. A previous diagnosis of colonic dysplasia (HR 3.34, 95% c.i. 1.01 to 10.97; *P* = 0.047), primary sclerosing cholangitis (HR 5.42, 95% c.i. 1.34 to 21.85; *P* = 0.018), and elective colectomy (HR 1.83, 95% c.i. 1.11 to 3.02; *P* = 0.018) was associated with an increased incidence of rectal cancer. Regarding endoscopic surveillance, there was a 43% decline in endoscopic procedures performed in 2020 (333 procedures) compared with 2019 (585 procedures).

**Conclusion:**

The incidence of rectal cancer after subtotal colectomy is low. Asymptomatic patients without evidence of rectal dysplasia should be carefully counselled on the possible benefits and risks of prophylactic proctectomy.

## Teaser

This study used observational data to look at the risk of rectal cancer after subtotal colectomy for IBD. The incidence of rectal cancer after subtotal colectomy for IBD is low. Asymptomatic patients should be carefully counselled on the risks of proctectomy, especially when the indication for surgery is prophylactic without evidence of rectal dysplasia.

## Introduction

Inflammatory bowel disease (IBD) is known to increase the risk of colorectal neoplasia. This is due to chronic inflammation causing DNA damage that results in the activation of pro-carcinogenic genes and the silencing of tumour suppressor genes in the colorectal mucosa^1^. Patients with IBD need clinicians to be able to accurately prognosticate their risk of developing such malignant complications over their lifetime^2^.

Although in many cases colectomy provides respite from the symptoms of colonic IBD, the dilemma of managing the rectal remnant remains, where the focus becomes surveillance and mitigation of the risk of rectal neoplasia. The patient and clinician are then confronted with a choice in the management of the rectal remnant: proctectomy alone, proctectomy with reconstruction (ileal pouch anal anastomosis or, less commonly, ileorectal anastomosis), or endoscopic surveillance. Understanding the incidence of rectal cancer over time is key for the patient to make an informed decision regarding future management.

The current literature on the risk of rectal cancer in the rectal remnant is dominated by reports of retrospective single-centre studies that are at risk of recall and selection bias^3^. There is also evidence of publication bias, with a predominance of high-prevalence studies in the literature^3^. There is the potential for lead-time bias or overdiagnosis bias in studies of patients with IBD, as they are more likely to undergo screening for colorectal cancer compared with the population at large^4^. Further complicating matters is how the COVID-19 pandemic has reduced the provision of endoscopic screening and surgery available for patients with IBD, and the long-term implications of this are unknown^5^.

The primary aim of this observational nationwide study was to estimate the cumulative incidence of rectal cancer in patients with IBD after colectomy at 5, 10, and 15 years. The secondary aims were to: quantify the risk factors associated with developing rectal cancer; estimate the cumulative incidences of death, proctectomy, and pouch surgery; and analyse how the COVID-19 pandemic has affected the surveillance of the rectal remnant.

## Methods

### Data source

The English National Health Service (NHS) Hospital Episode Statistics (HES) database has been previously described^6^. Briefly, the HES database is an administrative database containing demographic, diagnostic, procedural, and outcome data for all inpatient hospital episodes in England (except for private patients treated in private hospitals). Diagnostic coding is based on ICD-10 and procedural coding is based on the Office of Population Censuses and Surveys Classification of Interventions and Procedures version 4 (OPCS-4).

### Study population

The HES database was interrogated to identify patients with IBD who had undergone subtotal colectomy.

A cohort of patients undergoing subtotal colectomy between 1 April 2002 and 31 March 2014 was identified. Patients were included when admitted for an episode with an OPCS-4 code indicating colectomy in any procedure field and an ICD-10 code of inflammatory bowel in any diagnostic field (*Table S1*). This episode was designated as the index episode. Patients were classified as IBD unclassified (IBD-U) when they had both ulcerative colitis (UC) and Crohn’s disease (CD) diagnoses coded for in diagnostic fields for different episodes 2 years before the index episode. A history of primary sclerosing cholangitis (PSC) was identified with the ICD-10 code for cholangitis (K830) for any episode 2 years before the index episode or during the index episode. The same method was used to identify a history of colorectal dysplasia with ICD-10 codes (D126, D127, and D128). The admission method code was used to identify the acuity (emergency or elective) of the colectomy admission.

Patients with invalid or missing data for sex or age were excluded. Patients with a diagnosis of colorectal cancer during the index episode or 2 years prior were excluded. Patients diagnosed with rectal cancer within 30 days of the index episode were excluded. Patients who had an inconsistent prior procedure 2 years before their index colectomy were also excluded (*Table S1*). The study inclusion and exclusion criteria are detailed in *Table 1*.

**Table 1:** Study inclusion and exclusion criteria

Inclusion criteria	Exclusion criteria
Diagnosis of ulcerative colitis or Crohn’s disease during colectomy episode	Inconsistent coded operation 2 years before colectomy
Aged greater than or equal to 18 years	Colorectal cancer diagnosis in 2 years before colectomy
Colectomy episode between 1 April 2002 and 31 March 2014	Rectal cancer diagnosis within 30 days of index operation
Elective or emergency colectomy	Aged less than 18 years
	Invalid data recorded for age or sex
	Invalid data recorded for date of death or admission date

Patients were followed up in the database until an admission with a diagnosis of cancer in the rectal remnant, proctectomy (with or without pouch surgery) (*Table S1* reports specific ICD-10 and OPCS-4 codes), death, or the study end date (31 March 2021). The HES database was linked to Office for National Statistics (‘ONS’) data to capture deaths outside of hospital.

### Outcomes of interest

The first outcome of interest was the cumulative incidence of rectal cancer in patients with IBD after colectomy in IBD-U, UC, and CD, as well as analyses of the clinical variables associated with this outcome. The secondary outcomes were the cumulative incidences of death, proctectomy, and pouch surgery in these subgroups, as well as a sub-analysis of the frequency of endoscopic surveillance over a number of years, including during the COVID-19 pandemic.

### Statistical analysis

Patient characteristics analysed included age, sex, ethnicity, and area-level socio-economic deprivation. Ethnicity is described using six major groups (white, mixed, Asian, black, Chinese or other, and not known/not stated) and deprivation is described using the Index of Multiple Deprivation (quintiles 1–5, where 1 corresponds to the least deprived and 5 corresponds to the most deprived, with 6 being used when the level of deprivation is not known). Age, duration of follow-up, and disease-free survival are described using the median (interquartile range (i.q.r.)).

The cumulative incidence function was used to estimate the incidence of rectal cancer with proctectomy, pouch surgery, and death as competing events and plots were created. A Fine–Gray subdistribution hazard model^7^ was used to estimate the effects of covariates on the hazard of developing rectal cancer after accounting for competing events. This model was selected as it is more suited for estimating prognosis than a cause-specific hazard model^7^.

Where there are less than five patients in a particular reported group, the results are not detailed to prevent identification.

All statistical analyses and plots were performed and created using SAS version 9.4.

## Results

### Cumulative incidences of rectal cancer, death, proctectomy, and pouch reconstruction

**Fig. 1:**
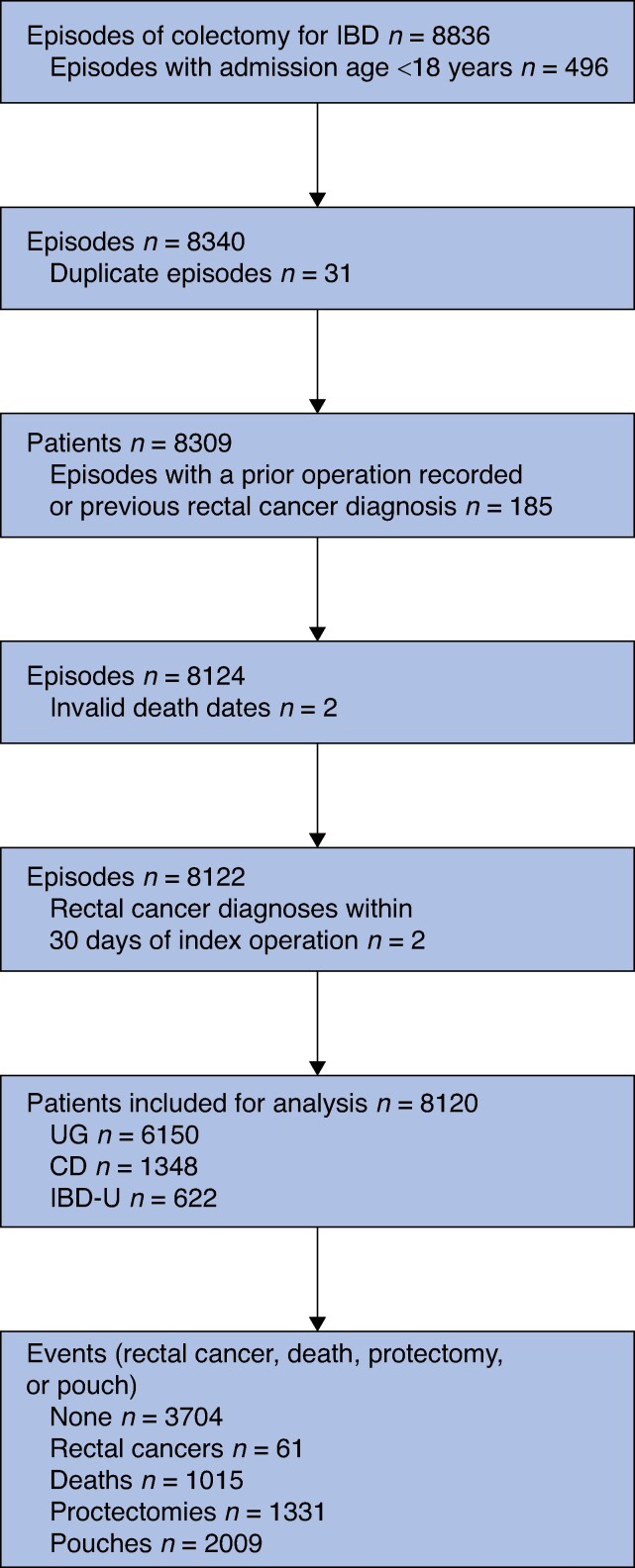
Flow chart of patients included in the present study UC, ulcerative colitis; CD, Crohn’s disease; IBD-U, inflammatory bowel disease unclassified.

A total of 8120 patients were eligible for inclusion in the study (*Fig. 1*) and the characteristics of the included patients are shown in *Table 2*. The median follow-up was 7.1 (i.q.r. 1–12) years and the median age at colectomy was 46.0 (i.q.r. 31–60) years. There were 1015 (12.5%) deaths during the study. A total of 61 patients (0.8%) were diagnosed with cancer in the rectal remnant, with a median disease-free survival of 7.5 (1.4–11.7) years. *Table 3* shows the characteristics of the patients who developed rectal cancer. A proctectomy alone was performed for 1331 patients (16.4%) and 2009 patients (24.7%) underwent pouch surgery.

**Table 2:** Characteristics of the 8120 patients included in the present study

Variable	Value
**Sex**	
Male	4518 (55.6)
Female	3602 (44.4)
**Age**	
Age at colectomy (years), median (i.q.r.)	46.0 (31.0–60.0)
**Diagnosis**	
UC	6150 (75.7)
CD	1348 (16.6)
IBD-U	622 (7.7)
**Ethnicity**	
White	6718 (82.9)
Mixed	40 (0.5)
Asian	370 (4.6)
Black	66 (0.8)
Chinese or other	89 (1.1)
Not known/not stated	1402 (1.0)
**Deprivation (IMD quintile)**	
1 (least deprived)	1515 (20.9)
2	1515 (20.9)
3	1470 (20.3)
4	1429 (19.7)
5 (most deprived)	1280 (17.7)
6 (not known)	911 (11.2)
**Follow-up**	
Follow-up time (years), median (i.q.r.)	7.1 (1.0–12.0)
**Risk factors**	
PSC	54 (0.7)
Colonic dysplasia	52 (0.6)
Rectal dysplasia	29 (0.4)
Colon cancer	94 (1.1)
**Year of colectomy**	
2002–2008, *n*	4223
2009–2014, *n*	3887
**Acuity of colectomy**	
Emergency	5207 (64.1)
Elective	2913 (35.9)

Values are *n* (%) unless otherwise indicated. i.q.r., interquartile range; UC, ulcerative colitis; CD, Crohn’s disease; IBD-U, inflammatory bowel disease unclassified; IMD, index of multiple deprivation; PSC, primary sclerosing cholangitis.

**Table 3:** Characteristics of the 61 patients who developed rectal cancer

Variable	Value
**Sex**	
Male	43 (70.5)
Female	18 (29.5)
**Age**	
Age at colectomy (years), median (i.q.r.)	48 (40–63)
**Diagnosis**	
UC	47 (77)
CD	7 (11.5)
IBD-U	7 (11.5)
**Ethnicity**	
White	47 (77.0)
Mixed	<5 (<8)
Asian	7 (11.5)
Black	<5 (<8)
Chinese or other	<5 (<8)
Not known/not stated	6 (9.9)
**Deprivation (IMD quintile)**	
1 (least deprived)	9 (16.4)
2	10 (18.2)
3	8 (14.6)
4	13 (23.6)
5 (most deprived)	15 (27.3)
6 (unknown)	6 (9.9)
**Disease free survival**	
Disease-free survival (years), median (i.q.r.)	7.5 (1.4–11.7)
**Risk factors**	
PSC	<5 (<8)
Colonic dysplasia	<5 (<8)
Rectal dysplasia	<5 (<8)
Colon cancer	<5 (<8)
**Acuity of colectomy**	
Emergency	30 (49.2)
Elective	31 (50.8)

Values are *n* (%) unless otherwise indicated. i.q.r., interquartile range; UC, ulcerative colitis; CD, Crohn’s disease; IBD-U, inflammatory bowel disease unclassified; IMD, index of multiple deprivation; PSC, primary sclerosing cholangitis.

**Fig. 2:**
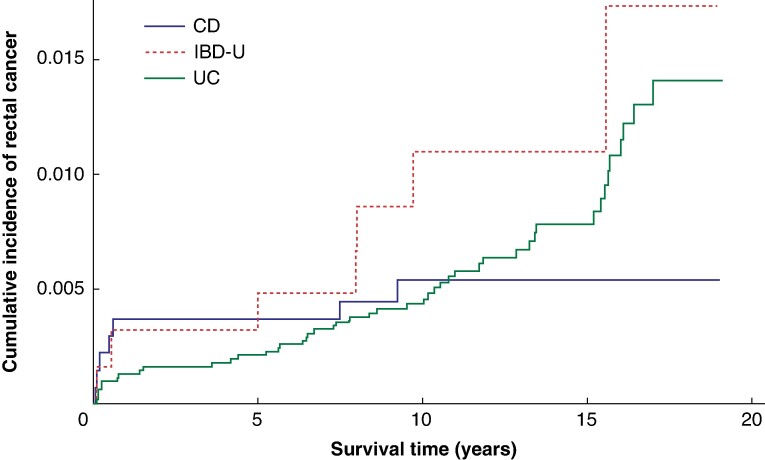
Cumulative incidence function plot for rectal cancer diagnosis CD, Crohn’s disease; IBD-U, inflammatory bowel disease unclassified; UC, ulcerative colitis.

The overall cumulative incidence of rectal cancer after colectomy was 0.26% (95% c.i. 0.17% to 0.39%), 0.49% (95% c.i. 0.36% to 0.68%), and 0.77% (95% c.i. 0.57% to 1.02%) at 5, 10, and 15 years respectively. The cumulative incidence plots for rectal cancer separated by diagnoses of UC, CD, and IBD-U are shown in *Fig. 2*. Gray’s test for equality did not find a significant difference in cumulative incidence (*P* = 0.316) amongst the three diagnoses.

**Fig. 3:**
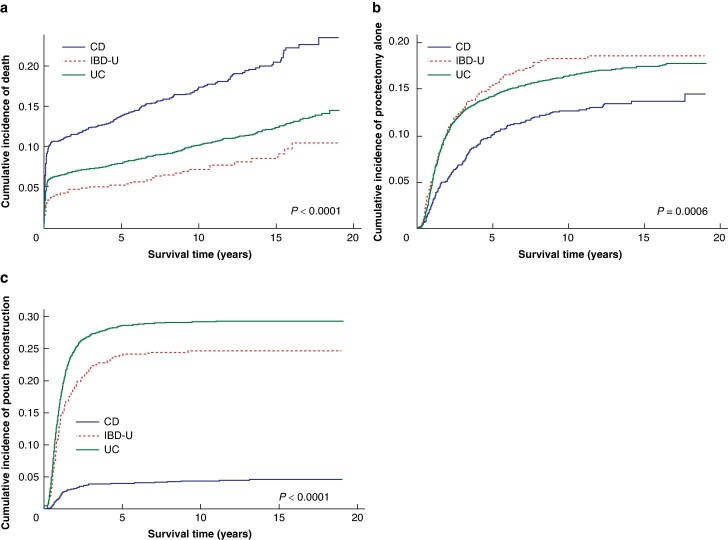
Cumulative incidence function plots for competing events (death, proctectomy alone, and pouch reconstruction) separated by diagnosis **a** Death (Gray’s test for equality, *P* < 0.0001). **b** Proctectomy alone (*P* = 0.0006). **c** Pouch reconstruction (*P* < 0.0001). CD, Crohn’s disease; IBD-U, inflammatory bowel disease unclassified; UC, ulcerative colitis.

The cumulative incidence plots for death, proctectomy alone, and pouch reconstruction are shown in *Fig. 3*. All three plots show significant differences in the cumulative incidences of these events amongst patients with UC, CD, and IBD-U. Patients with CD had a lower 10-year cumulative incidence of proctectomy after colectomy (12.7% (95% c.i. 10.9% to 14.5%)) than patients with UC (16.4% (95% c.i. 15.5% to 17.3%)) and IBD-U (18.2% (95% c.i. 15.4% to 21.5%)) (*P* = 0.0006). Patients with CD also had the lowest 10-year cumulative incidence of pouch reconstruction (4.3% (3.3% to 5.5%)) compared with patients with UC (29.2% (95% c.i. 28.1% to 30.3%)) and IBD-U (24.7% (95% c.i. 21.3% to 28.2%)) (*P* < 0.0001).

Patients with CD had a higher 10-year cumulative incidence of death (17.3% (95% c.i. 15.3% to 19.4%)) than patients with UC (10.3% (95% c.i. 9.5% to 11.1%)). Patients with IBD-U had the lowest cumulative incidence of death (7.2% (95% c.i. 5.4% to 9.5%)) of all (*P* < 0.0001). Most of this difference stems from differential mortality in the postoperative interval after colectomy, as shown in *Fig. 3*.

### Subdistribution hazard model for rectal cancer

The HRs and associated 95% confidence intervals for covariates for the subdistribution hazard model for a rectal cancer diagnosis are shown in *Table 4*. A previous diagnosis of colonic dysplasia, PSC, and elective colectomy were all associated with an increased incidence of rectal cancer. Female sex was associated with a 45% lower risk of rectal cancer. There was weaker evidence for an association between older age and the incidence of rectal cancer. Type of IBD diagnosis and year of colectomy were not associated with the cumulative incidence of rectal cancer. Results from the cause-specific hazard model were similar and are shown in *Table S2*.

**Table 4:** HRs and associated 95% confidence intervals for covariates for the subdistribution hazard model for a rectal cancer diagnosis

Variable	HR (95% c.i.)	*P*
Age (per year increase)	1.01 (1.00,1.03)	0.057
**Sex**		
Male	1	
Female	0.55 (0.31,0.95)	0.034
**IBD diagnosis**		
UC	1	
CD	0.78 (0.35,1.75)	0.549
IBD-U	1.55 (0.69,3.44)	0.287
PSC	5.42 (1.34,21.85)	0.018
Colonic dysplasia	3.34 (1.01,10.97)	0.047
**Acuity of colectomy**		
Emergency	1	
Elective	1.83 (1.11,3.02)	0.018
Year of colectomy (per year increase)	0.93 (0.86,1.01)	0.069

IBD, inflammatory bowel disease; UC, ulcerative colitis; CD, Crohn’s disease; IBD-U, inflammatory bowel disease unclassified; PSC, primary sclerosing cholangitis.

### Trends in endoscopic surveillance of the rectal remnant over a number of years, including during the COVID-19 pandemic

**Fig. 4:**
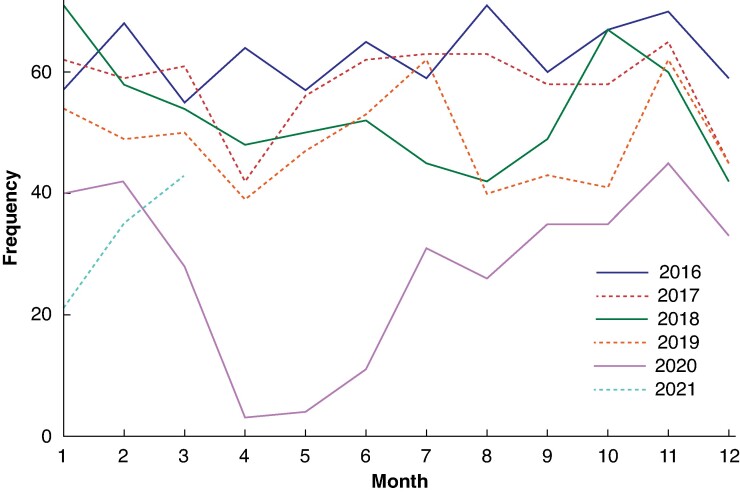
Frequency of endoscopic surveillance of the rectal remnant from January 2016 to March 2021

*Figure 4* shows the trends in endoscopic surveillance amongst patients who had a colectomy in this study from January 2016 to March 2021. There is a trend towards fewer endoscopic procedures with each passing year for this cohort. Even allowing for this, 2020 shows a marked decrease in the trend compared with the previous 4 years. There were 333 endoscopic procedures performed in 2020 compared with 585 in 2019, representing a 43% decline in surveillance.

## Discussion

This large observational study investigating rectal cancer risk after colectomy for IBD shows that the 10-year cumulative incidence is low (0.49%). This study did not find a significant difference in the incidence of cancer across the three diagnoses; however, it shows that men and those diagnosed with PSC or colonic dysplasia are at increased risk of developing rectal cancer. Finally, it shows the challenges in England during the COVID-19 pandemic, with endoscopic surveillance declining 43% in 2020 compared with 2019.

A systematic review and meta-analysis of the rectal cancer risk after colectomy for IBD found that most studies assessing risk in the rectal remnant were retrospective single-centre cohort studies with insufficient sample sizes to calculate prevalence^3^. The studies included were at high risk of selection and recall bias. There was evidence of publication bias, with low-prevalence studies missing from the literature^3^. The present study, which suggests a lower prevalence than the single-centre retrospective cohort studies, does fill that hypothesized publication gap.

Comparing the present findings with the previous largest observational studies, a comparable incidence of rectal cancer is found. A previous investigation followed a cohort of 5886 patients who underwent colectomy for UC in Sweden^8^. They found that the cumulative risk of rectal remnant cancer was 0.5% at 10 years and 2.2.% at 20 years based on data from 4358 patients^8^. Another investigation performed a retrospective cohort study of 3700 patients with IBD in Ontario, Canada, with a median follow-up of 4.3 years. They did not find a difference in the risk of rectal cancer between UC and CD patients. They found a cumulative incidence of rectal cancer of 0.81% and 1.86% at 10 and 20 years respectively. Finally, a Danish study found a 0.1% and 1.9% risk of rectal cancer at 10 and 35 years of follow-up respectively amongst 4703 patients with IBD^9^. They did not find a significant difference in the standardized incidence ratio (‘SIR’) for those with UC or CD.

The subdistribution hazard model presented in this research found that PSC and colonic dysplasia were associated with an increased incidence of rectal cancer, as has been previously demonstrated^8,10^. Male sex has sometimes been shown to be associated with rectal cancer^10^ and the present study adds to this evidence.

It was also demonstrated that elective colectomy is associated with an increased incidence of cancer. The causal mechanism for this is not completely clear. Patients who undergo elective rather than emergency colectomy may have had a longer duration of disease and therefore be at greater risk of neoplastic change. The indication for surgery is more likely to be dysplasia and suspected cancer in elective cases. Those who have undergone emergency colectomy may have had a shorter duration of more severe colitis that is treated surgically at an earlier stage^11,12^.

Secondary to the main findings, it was also shown that postoperative mortality is increased for patients with CD undergoing colectomy. This has been shown previously by others^13,14^. The difference is so marked it can still be seen a decade later in the cumulative incidence. This study provides further evidence that CD patients undergoing colectomy are at higher risk of death and those diagnosed with both UC and CD tend to have the lowest risk of all. Why this differential mortality occurs is not clear and should be the focus of future research. Possible causes of this differential mortality might be that patients with CD present with fistula and may have a more marked septic response.

Guidelines from the British Society of Gastroenterology for IBD recommend that surveillance begins at 8 years after diagnosis or the start of symptoms, with the surveillance interval being influenced by the extent of the disease, the level of inflammation, family history, the presence of dysplasia, and a diagnosis of PSC^15^. Surveillance of the rectal remnant is recommended^15^. A previous report proposed a post-colectomy surveillance algorithm that takes into account duration of diagnosis, colorectal dysplasia, PSC, and a history of colorectal cancer, separating patients into low-, intermediate-, or high-risk surveillance intervals of 5 years, 2–3 years, or 1 year respectively^16^. Another study showed that surveillance endoscopy was associated with a 68% reduction of the sub-hazard of rectal cancer^17^.

The present study shows that surveillance of the rectal remnant was affected by the COVID-19 pandemic, with a 43% reduction in the number of endoscopies in 2020 compared with 2019. How surveillance programmes will recover after the pandemic and whether the reduced surveillance has had any effect on patient outcomes are yet to be seen.

This study will help counsel patients for completion proctectomy (with or without pouch reconstruction) or ongoing surveillance. Completion proctectomy is a major operation, with associated morbidity. Clear indications for completion proctectomy for IBD are symptomatic proctitis, high-grade dysplasia, anal stricture, or unmanageable fistulating disease^18^. However, the decision is not clear for patients who are asymptomatic, without dysplasia, and those with a good quality of life with an end ileostomy. It is known that pelvic and perineal surgery carries specific risks. Pouch surgery is associated with decreased fertility in females, which was confirmed in a recent systematic review^19^. Female patients take longer to conceive and are more likely to use *in vitro* fertilization after restorative proctocolectomy^20^. This is likely due to adhesion formation in the female pelvis after surgery^21^. With regards to function, a prospective cohort study found significant improvement in sexual function for men after proctectomy, with a less marked improvement for women^22^. Furthermore, wound complications can be common, for example persistent perineal sinus, especially in those with CD—the incidence may be close to 50% at 1 year, according to a retrospective cohort study^23^.

There are limitations to this study. This study relies on routinely collected administrative data and the reliability of coded data. The reliability of some key elements of HES data has previously been demonstrated in other studies^24–26^. However, there is no specific code for cancer in the retained rectum. It may be coded as a malignant neoplasm of the rectum or as a malignant neoplasm of the large intestine not otherwise specified or as a malignancy where the origin cannot be easily classified. The present study also used secondary diagnostic codes to look for diagnoses of PSC, colonic dysplasia, or rectal dysplasia, and generally these were underutilized codes, with all having a prevalence of less than 1% in the cohort. These low prevalences explain the wide confidence intervals associated with their HRs and therefore the size of the HRs should not be considered accurate. This study relies upon a diagnosis of cancer being made during a hospital admission, but cancer diagnoses can be made when patients are outpatients and so the cumulative incidences could be underestimates. This could be improved by linkage to cancer registration data in a future study. Also, the present study estimates incidences up to 15 years after colectomy. Many patients with IBD have a colectomy in early life and the authors are unable to comment on lifetime risks for such young patients. Finally, some medications are not coded or coded with limitations (only certain biological therapies delivered as outpatient infusions) in the HES database.

The strength of this study is in the standardized capture of demographic, diagnostic, and procedural data of all inpatient stays in the HES database. This study is not susceptible to the recall and selection bias of single-centre or multicentre retrospective cohort studies. It captures patients who have moved from one region of England to another and would have been lost to follow-up in a single-centre study. This is reflected in the relatively low cumulative incidence in this study and other nationwide studies compared with the single-centre retrospective series that dominate the literature.

This study shows that the 10-year risk of rectal cancer after colectomy is low (0.49%). All patients should be offered endoscopic surveillance to ensure rectal cancer is not missed. Patients at increased risk of rectal cancer, such as those with PSC, those with dysplasia, and men, should be offered surveillance at shorter intervals. The type of underlying IBD should not affect surveillance. Surveillance programmes have been interrupted by the COVID-19 pandemic and the long-term effect of this is yet to be seen. Asymptomatic patients with a good quality of life with an end ileostomy should be carefully counselled on the risks of proctectomy, especially when surgery is prophylactic without evidence of rectal dysplasia.

## Supplementary Material

zrae074_Supplementary_Data

## Data Availability

The pseudonymized patient data that were used for this study can be accessed by contacting NHS Digital, now part of NHS England (see https://digital.nhs.uk/services/data-access-request-service-dars). Access to these data is subject to a data sharing agreement (‘DSA’) containing detailed terms and conditions of use after protocol approval from NHS Digital.
