# Supplementary material for: Incidence of rectal cancer after colectomy for inflammatory bowel disease: nationwide study
Source: BJS Open. 2024 Oct 15;8(5):zrae074. doi: 10.1093/bjsopen/zrae074 (PMC11474238; doi:10.1093/bjsopen/zrae074)
Supplement: zrae074_Supplementary_Data [file zrae074_supplementary_data.docx]

**The incidence of rectal cancer after colectomy for inflammatory bowel disease: a nationwide study**

Mohammed Deputy1,2, Guy Worley1,2, Elaine M Burns1,2, Alex Bottle3, Paul Aylin3, Ailsa Hart2,4, Omar Faiz1,2

1. Surgical Epidemiology, Trials and Outcome Centre, St Mark’s Hospital and Academic Institute
2. Department of Surgery and Cancer, Imperial College London
3. School of Public Health, Imperial College London
4. Department of Gastroenterology, St. Mark’s Hospital and Academic Institute

**Corresponding author:** Mohammed Deputy, Surgical Epidemiology, Trials and Outcome Centre, St Mark’s Hospital and Academic Institute, Watford Rd, Harrow, HA1 3UJ, m.deputy@nhs.net

**Data Availability Statement:** The pseudonymised patient data that were used for this study can be accessed by contacting NHS Digital, now part of NHS England (see https://digital.nhs.uk/services/data-access-request-service-dars). Access to these data is subject to a data sharing agreement (DSA) containing detailed terms and conditions of use following protocol approval from NHS Digital.

**Conflicts of Interest**: None

**Supplementary Materials - Index**

| **Supplementary Tables** |  |
| --- | --- |
| Supplementary Table 1 | *pag. 2* |
| Supplementary Table 2 | *pag. 3* |

**Supplementary Table 1: ICD-10 and OPCS-4 codes**

**ICD-10 codes**

UC

K510 K512 K513 K515 K518 K519

CD

K501 K508 K509

Rectal cancer

C190 C200 C218

Colon cancer

C180 C181 C182 C183 C184 C185 C186 C187 C188 C189

**OPCS-4 Codes**

Colectomy

H052 H053 H058 H059 H114 H115 H298 H299

Pouch surgery

H042 H043 H291 H292 H293 H294 G725

Completion proctectomy

H041 H048 H335 H336 H337 H338 H339

Previous inconsistent operation codes excluded (if in 2 years prior to colectomy)

H041 H042 H043 H048 H049 H051 H052 H053 H058 H059 H111 H112 H113 H114 H115

H291 H292 H293 H298 H299 H331 H332 H334 H335 H336 H337 H338 H339

**Supplementary Table 2**

| **Variable** |  | **Hazard Ratio** | **95% Confidence Limits** | **P value** |
| --- | --- | --- | --- | --- |
| Age (per year increase) |  | 1.011 | 0.997 - 1.027 | 0.1325 |
|  |  |  |  |  |
| Sex | Male | 1.000 |  |  |
|  | Female | 0.536 | 0.307 - 0.935 | 0.0281 |
|  |  |  |  |  |
| IBD diagnosis | UC | 1.00 |  |  |
|  | CD | 0.619 | 0.277 - 1.382 | 0.5488 |
|  | IBD-U | 1.414 | 0.635 - 3.147 | 0.3966 |
|  |  |  |  |  |
| PSC |  | 5.181 | 1.240 - 21.654 | 0.0242 |
|  |  |  |  |  |
| Colonic dysplasia |  | 2.792 | 0.857 – 9.094 | 0.0884 |
|  |  |  |  |  |
| Acuity of colectomy | Emergency | 1.000 |  |  |
|  | Elective | 1.696 | 1.020 – 2.820 | 0.0419 |
|  |  |  |  |  |
| Year of colectomy (per year increase) |  | 0.989 | 0.907 - 1.078 | 0.7977 |

Hazard ratios and 95% confidence limits from cause-specific hazard model for rectal cancer diagnosis. UC – ulcerative colitis, CD – Crohn’s disease, IBD-U – inflammatory bowel disease unspecified, PSC – primary sclerosing cholangitis
